# Epidemiology of two human protoparvoviruses, bufavirus and tusavirus

**DOI:** 10.1038/srep39267

**Published:** 2016-12-14

**Authors:** Elina Väisänen, Minna Paloniemi, Inka Kuisma, Väinö Lithovius, Arun Kumar, Rauli Franssila, Kamruddin Ahmed, Eric Delwart, Timo Vesikari, Klaus Hedman, Maria Söderlund-Venermo

**Affiliations:** 1 Department of Virology, University of Helsinki, Helsinki 00290, Finland; 2 Vaccine Research Center, University of Tampere, Tampere 33520, Finland; 3 Fimlab laboratories ltd, Tampere 33520, Finland; 4 Health Sciences North Research Institute, Sudbury, ON P3E 5J1, Canada; 5 Department of Pathobiology and Medical Diagnostics, Faculty of Medicine, Universiti Malaysia Sabah, Kota Kinabalu 88400, Sabah, Malaysia; 6 Blood Systems Research Institute, San Francisco, CA 94118, USA; 7 Department of Laboratory Medicine, University of California at San Francisco, San Francisco, CA 94118, USA; 8 Helsinki University Hospital, HUSLAB, Helsinki 00290, Finland

## Abstract

Two human parvoviruses were recently discovered by metagenomics in Africa, bufavirus (BuV) in 2012 and tusavirus (TuV) in 2014. These viruses have been studied exclusively by PCR in stool and detected only in patients with diarrhoea, although at low prevalence. Three genotypes of BuV have been identified. We detected, by in-house EIA, BuV1-3 IgG antibodies in 7/228 children (3.1%) and 10/180 adults (5.6%), whereas TuV IgG was found in one child (0.4%). All children and 91% of the adults were Finnish, yet interestingly 3/6 adults of Indian origin were BuV-IgG positive. By competition EIA, no cross-reactivity between the BuVs was detected, indicating that the BuV genotypes represent distinct serotypes. Furthermore, we analysed by BuV qPCR stool and nasal swab samples from 955 children with gastroenteritis, respiratory illness, or both, and found BuV DNA in three stools (0.3%) and for the first time in a nasal swab (0.1%). This is the first study documenting the presence of BuV and TuV antibodies in humans. Although the seroprevalences of both viruses were low in Finland, our results indicate that BuV infections might be widespread in Asia. The BuV-specific humoral immune responses appeared to be strong and long-lasting, pointing to systemic infection in humans.

The development of modern deep sequencing and metagenomic techniques have facilitated recent discoveries of several novel parvoviruses in various animal species, including humans. Of the novel putative human parvoviruses, bufavirus (BuV) and tusavirus (TuV) are among the newest: both of these viruses were originally discovered in the stools of diarrheic children in Africa - BuV in 2012 in Burkina Faso and TuV in 2014 in Tunisia^1^,^2^.

Parvoviruses are small, non-enveloped viruses with a single-stranded DNA genome of 4–6 kb, encoding only a few proteins. The classification of parvoviruses is currently based on the non-structural protein (NS1) sequence^3^, and both BuV and TuV are classified in the *Protoparvovirus* genus in the *Parvoviridae* family. Human bufaviruses are conserved in the NS1 protein (94–96% similarity at the amino acid (aa) level)^4^, and thus belong to one species, *Primate protoparvovirus 1*^3^. However, the BuV capsid proteins VP1 and VP2 share only 71–78% and 64–73% similarities at the aa level, respectively, generating three genotypes^4^,^5^. Tusavirus on the other hand has been described only in one child^2^, although partial TuV-like sequences have been detected in fur seals in Brazil^6^.

To date, bufavirus has been searched for exclusively in stool samples, and it has been detected in diarrheal stools of children and adults in Africa, Europe and Asia, including Burkina Faso, Tunisia, Bhutan, Finland, the Netherlands, Thailand, Turkey and China^1^,^5^,^7^,^8^,^9^,^10^,^11^. The prevalence in patients has been low, 0.3% to 4%, and although in three publications stool samples from non-diarrheic patients or healthy subjects were shown to be BuV-DNA negative^9^,^10^,^11^, the etiological role of the virus in diarrhoea or in other human diseases remains uncertain.

BuV-like viruses have been found in wild and captive non-human primates as well as in swine, shrews, rats, bats and fur seals^4^,^6^,^12^,^13^,^14^,^15^,^16^,^17^. The detection of these viruses in sera of rhesus monkeys in the USA, and in the spleen of wild baboons and shrews in Zambia, suggests that BuV-like viruses may cause systemic infections^12^,^13^.

We first analysed by quantitative PCR the presence of BuV DNA in stool and, for the first time, also in nasal swab samples from 955 children with symptoms of acute gastroenteritis, acute respiratory infection or both. The mere presence of viral DNA in stool or in other sample types does, however, not necessarily indicate a causal role to a disease, neither does the transient PCR positivity give a true picture of how common the virus is in the population. In this study we therefore investigated the prevalence of BuV- and TuV-specific IgG antibodies, which can reveal prior virus encounters. We created virus-like particles (VLP) of the major VP2 capsid proteins of all three BuV genotypes and of TuV, applied them as antigens in IgG EIAs, and analysed 180 serum samples from healthy adults and 228 serum samples from the same paediatric cohort to assess the epidemiology of these viruses.

## Results

### Bufavirus DNA in nasal swab and stool samples in the paediatric cohort

BuV DNA was detected in 1/955 (0.1%) nasal swab and in 3/955 (0.3%) stools (Table 1). The results were confirmed by sequencing the qPCR amplicons. We were not able to amplify the VP region of the viral genome from these samples, possibly due to low quantity and/or limited sample volume; the genotypes therefore remained unknown. The viral loads of the samples were 2.1 × 10^2^–1.6 × 10^3^ copies/ml stool suspension and 4.6 × 10^3^ copies/ml nasal swab medium (Table 1). All four BuV DNA-positive children had gastrointestinal symptoms, while all of the 545 non-diarrheic children were BuV-DNA negative. However, all four children had in stool also another virus known to cause gastroenteritis (Table 1).

### Bufavirus and tusavirus VP2 VLPs

**Figure 1:**
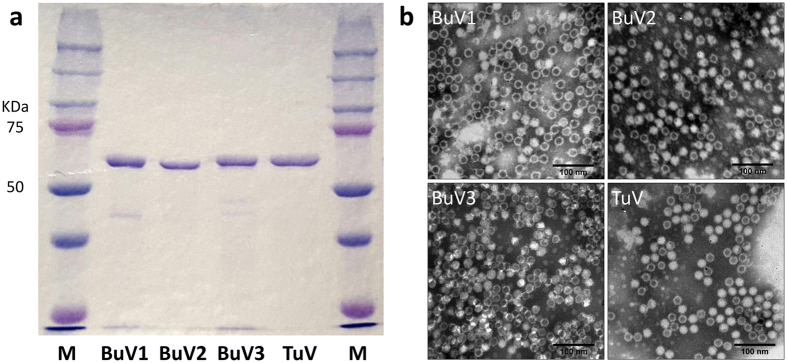
SDS-PAGE (**a**) and electron microscopy pictures (**b**) of VP2-VLPs of BuV1-3 and TuV.

The *VP2* genes were cloned from the original BuV and TuV DNA-positive stool samples and used to create recombinant VP2 VLPs for BuV1, 2 and 3 as well as TuV. The four types of VLPs were expressed in insect cell cultures and purified by CsCl ultracentrifugation. SDS-gel electrophoresis showed proteins of expected sizes, approximately 64 kDa for BuVs and 62 kDa for TuV, and electron microscopy disclosed parvovirus-like VLPs of ~25 nm in diameter (Fig. 1).

### Bufavirus and tusavirus IgG in adults

**Figure 2:**
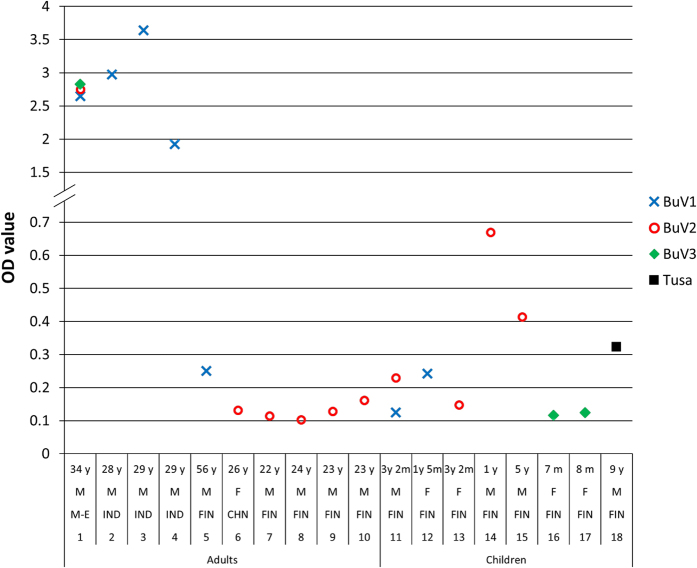
BuV and TuV IgG-positive adults and children. The figure presents the EIA OD values from the samples. Blue x marks the BuV1 IgG level in the sample, red circle the BuV2 IgG level, green diamond the BuV3 IgG level and the black square the TuV IgG level. Below each result the following information is presented: Age, gender, geographical background (M-E = Middle-East, IND = India, CHN = China, FIN = Finland), case number and cohort.

Ten subjects (10/180, 5.6%) were BuV IgG seropositive: four subjects for BuV1, five for BuV2 and one for all three bufaviruses (triple positive) (Tables 2 and 3, and Fig. 2). Three of the BuV1 seropositives and the triple seropositive showed very high OD values (OD 1.9–4.0, Table 2 and Fig. 2). None of the 180 adult sera were positive for TuV IgG. Although most of the adults were of Finnish descent (163/180, 90.6%), of the ten BuV IgG-positive subjects five were from the Middle-East, India or China, including all four with high OD values. They had moved to Finland as adults, and were 26 to 34 years of age at the time of sampling. Consequently, the seroprevalence among the native Finnish subjects was 3.1% (5/163), whereas 5/12 (41.7%) Asian subjects and 3/6 (50%) Indian subjects were seropositive. All BuV IgG-positive adults of Finnish background had low OD values.

Follow-up samples were available from five BuV IgG-positive adults, including all four with high OD values. The samples were analysed with BuV-IgG EIA, and the OD values, which reflect the antibody titres in the tested subjects, were shown to remain stable for up to six years (Table 2).

### Bufavirus and tusavirus IgG in children

Among the 228 children studied for BuV and TuV IgG antibodies, seven (3.1%) had BuV IgG and one (0.4%) had TuV IgG. Of the BuV IgG-positive cases, one had IgG for BuV1, three for BuV2, two for BuV3 and one for both BuV1 and 2 (Fig. 2). The BuV-seropositive children were between 7 months and 5 years of age at the time of sampling, whereas the TuV-seropositive child was 9 years of age.

In addition to the true BuV or TuV seropositive samples, we encountered four children with low BuV IgG OD values with indeterminate blocking results (one BuV1 and three BuV2). These results were interpreted as negative.

Among the BuV DNA-positive cases, a serum sample was available only from the nasal swab-positive child. The serum was both BuV-DNA and BuV-IgG negative for all BuV types pointing either to a superficial or local respiratory tract infection without viremia or antibody responses or to a very early acute systemic infection still awaiting an immune response. Although this 20-month-old child had been hospitalized for four days and had underlying health issues including operated cleft palate, heart problems and asthma, a serologically confirmed acute HBoV2 infection suggests that this child was B-cell immunocompetent^18^.

### Specificity of bufavirus and tusavirus B-cell immunity

In order to ensure that the EIA absorbance values were specific for the particular virus, i.e. not due to cross-reactivity with known similar viruses or unspecific reactions, all samples with a measurable OD value of ≥0.1 underwent confirmatory competition assays by blocking with soluble VLPs and insect cell lysate. Samples with OD values below 0.1 were too difficult to unambiguously confirm by blocking and were thus considered negative. Of note, the determination of an exact cutoff value was not crucial for the interpretation of the results as all samples with OD values of ≥0.1 were confirmed by competition assay and only the confirmed results were considered genuinely positive.

The antibodies in all BuV- or TuV-seropositive samples showed no cross-reactivity between the BuV genotypes or TuV, including the triple-BuV seropositive plasma (Case 1; Table 3). Both in a high-positive adult (Case 1) and in a low-positive adult (Case 5), we could show that the BuV antibody levels and specificity remained constant for at least six years (Table 2). Furthermore, among the 84 medical students, the BuV1-3 and TuV IgG EIA results correlated neither with each other nor with the previously analysed IgG EIA results of six other human parvoviruses: HBoV1-4, PARV4, or parvovirus B19 (data not shown)^19^,^20^,^21^.

Notably, some of the sera did contain antibodies toward High5 insect cells in which our antigens were expressed: 9/180 (5.0%) in the adult cohort with ODs of 0.104–0.616, and 13/228 (5.7%) in the paediatric cohort with ODs of 0.096–0.404. Some of these High5 backgrounds would have yielded incorrect positivity in the unblocked BuV IgG EIAs, more with BuV2 and 3 than BuV1 or TuV antigens. However, we were able to distinguish false from genuine reactivity by competing with insect cell lysate, as can be seen in patient number 4 (Tables 2 and 3): the BuV2 and BuV3 reactivities were completely blocked in the competition assay by insect cells, but less or not at all by VLPs of either of these two BuVs, whereas the BuV1 result was blocked with the homologous BuV1 (Table 3). In our previous studies we have seen the same phenomenon with Sf9 insect cells. Human antibodies towards insect cell components should therefore be considered when applying in EIA insect cell-derived antigens.

## Discussion

Bufavirus and tusavirus are among the newest parvoviruses discovered in humans by deep sequencing^1^,^2^. Until now, human bufaviruses, mostly BuV1 or BuV3, has been detected by PCR in several geographical locations in Africa, Europe and Asia; but exclusively in diarrheal stools^1^,^5^,^7^,^8^,^9^,^10^,^11^. Studies have not included other sample types or other diagnostic means, yet the closely related BuV-like viruses have been found in spleens or sera of infected animals, indicating systemic infections^12^,^13^. Here we analysed for BuV DNA, stool samples and nasal swabs; as well as for BuV and TuV antibodies, sera of children with respiratory infection, gastroenteritis or both. We also determined the corresponding seroprevalences among asymptomatic adults.

In the stool samples, we found a low BuV-DNA prevalence in Finnish children presenting with acute gastroenteritis with or without respiratory tract infection, in line with prior reports^1^,^5^,^9^,^10^,^11^. We observed for the first time BuV DNA in a nasal swab, albeit at a very low quantity, and with no BuV IgG nor DNA detectable in a corresponding serum. In each child with BuV DNA in stool, a known gastrointestinal virus was identified, possibly accounting for the illness. However, none of the 545 children without gastroenteritis had BuV DNA in stool, suggesting BuV could be a contributing factor to the disease.

Human parvovirus VLPs can be constructed using solely the major capsid protein, and such VLPs are often the antigens of choice in serodiagnosis^19^,^22^,^23^,^24^,^25^,^26^,^27^. In this study we showed that the VP2 proteins of all three BuV genotypes and TuV are capable of assembling into VLPs, as with other human parvoviruses, and used these VLPs in EIAs for BuV and TuV serology.

The highest BuV IgG prevalence was detected among Asian asymptomatic adults; however, without statistical power due to low sample number. Of the Asian subjects 5/12 were positive, and the highest number was observed among Indian persons of whom 3/6 were BuV-IgG positive. These individuals had also the highest BuV IgG OD values, unlike the children or adults of Finnish descent. In addition, the prevalence of BuV antibodies in Finnish children (3.1%, 7/228) was identical to that in the native Finnish adults (3.1%, 5/163) raising the question why the BuV IgG prevalence did not increase with age. Although we did show that the antibody levels among adults can remain constant for at least six years, waning of the antibodies over time might play a role. Furthermore, if the primary infection of the host is local and superficial, it might not induce a strong long-lasting antibody response. With HBoVs it has been shown that exposure to homo- or heterologous HBoV species may boost the antibody response^28^,^29^. Thus, the similar BuV seroprevalence among Finnish adults and children might be a consequence of the general rarity of BuV infections in the Nordic countries, leading to inadequate maintenance of antibody responses.

In our previous study of stools of Finnish children and adults during 2012–2013, we detected BuV DNA exclusively of genotype 1^7^. In the present study we found six children with antibodies to genotypes 2 or 3, while only two had antibodies to genotype 1 (of note, one child had both BuV1 IgG and BuV2 IgG). These BuV2 or 3 IgG-positive children were 7 months to 5 years of age at the time of sampling (2009 to 2011), suggesting that BuV2 and maybe BuV3 might have circulated in Finland between 2006 and 2011. Unfortunately, we do not have travel information of these children. The BuV3 antibodies in the two 7- to 8-month-old infants might be maternal. It is interesting that in both of our cohorts BuV2 IgG was the most frequent, while BuV2 DNA has been detected in only a single patient, in the original publication^1^.

Most of the human parvoviruses have several variants or genotypes. This is the case also for BuV. The variants of human parvovirus B19 (B19V) differ in VP2 aa sequence by only 1–2%^30^,^31^, and cross-react fully in EIA. Therefore, B19V serodiagnosis can be done reliably using as antigen VP2 VLPs of any of the variants^32^,^33^,^34^. On the other hand, the more divergent four HBoVs (with a 11–23% difference in the major capsid protein aa sequence) are partly cross-reactive in EIA^19^. The cross-reactivity can be visualized by blocking the serum with heterologous HBoVs prior to the EIA. To identify such cross-reactivity is a prerequisite for correct interpretation of the HBoV EIA results^19^,^29^. The human bufavirus genotypes differ by 27–36% in VP2^4^, and in our EIA analysis the BuV genotypes were not cross-reactive. Neither the BuV genotype, the antibody level in the sample, nor the age of the subject affected assay specificity. Interestingly, we found one adult and one child with specific antibodies towards more than one BuV type (case 1 with a triple and case 7 with a double positivity, shown in Fig. 2). In conclusion, we showed the three BuV genotypes also to represent three diverse serotypes.

We analysed the serum IgG reactivities against a number of factors known to cause background in EIA, including insect cell lysate, non-antigen-coated microwells and streptavidin. Of both adults and children 5–6% had antibodies towards insect cell constituents, which would cause false positivity without competition assays. Such false reactions should be considered when applying insect-cell produced antigens in EIA.

This is the first published human study of TuV since the initial article. While we found a single child to exhibit TuV IgG, in confirmatory blocking a 5-fold higher concentration of the homologous antigen was required than in the corresponding BuV EIAs, to block the IgG binding. As none of the other antigens (BuV1-3) or insect cell lysate blocked the TuV IgG reactivity, the result was considered TuV specific. More studies are nevertheless needed to confirm whether TuV actually is a human virus.

Taken together, we observed for the first time BuV DNA, aside from pediatric stools, in a nasal swab. More importantly, this is the first study documenting the presence of BuV and TuV IgG antibodies in humans. We furthermore showed that the three bufaviruses represent different serotypes: none of the BuVs nor TuV were mutually cross-reactive in the cohorts studied. While in Finland the seroprevalences of both of these protoparvoviruses were low, we observed both higher OD values and a higher seroprevalence of BuV among persons with an Asian origin, indicating that BuV infections might be more common in other countries, particularly in India. The BuV-specific immune responses of all three serotypes appeared to be strong and lasting, pointing to systemic infections. Overall, our results together with those of others indicate that BuV is a human virus, whereas additional studies are needed to assess the host tropism of TuV and the clinical correlates and global epidemiology of these viruses as well as their transmission route(s).

## Materials and Methods

### Patients and Samples

The adult cohort consisted of serum or plasma samples from 180 healthy adult volunteers: 84 medical students from the University of Helsinki and 96 staff members from the Helsinki University Hospital or from the Faculty of Medicine, University of Helsinki (median age 30 years, range 19–65). A written informed consent was obtained from all subjects, and the samples were collected between 2007 and 2013. All medical students and 79/96 of the staff were of Finnish descent, the remaining 17 staff members were originally from Asia (12), the Americas (1) or other European countries (4). Follow-up samples were collected from BuV IgG-positive participants, or obtained (frozen-stored) from our archive. The Ethics Committee of Helsinki and Uusimaa Hospital District approved the study protocol, and the study was conducted in accordance with the relevant guidelines and regulations.

The samples and clinical information of the paediatric cohort have been described previously^18^. Shortly, the patients (n = 955) were recruited between September 2009 and August 2011 in Tampere, Finland, and the samples were collected throughout the year. The children were divided in three groups based on signs and symptoms: acute gastroenteritis (AGE, n = 172), acute respiratory tract infection (ARTI, n = 545) or symptoms of both (n = 238). The median age was 14 months (range 6 days – 15.6 years). There were patients both from a paediatric outpatient clinic, 137 (14.3%), and a hospital ward, 818 (85.7%). Stool and nasal swab samples were available from every child and serum samples were from 228 children of whom blood sampling was medically indicated. A written informed consent was obtained from the parents of all the children enrolled. The study was approved by the Ethics Committee of Pirkanmaa Hospital District, and, and the study was conducted in accordance with the relevant guidelines and regulations.

### DNA extraction and bufavirus quantitative PCR

DNA was extracted from stool and nasal swab samples as described in Paloniemi *et al*., and the DNA aliquots were analysed with BuV-qPCR as described in Väisänen *et al*.^7^,^18^. In qPCR-positive cases the amplicon was sequenced to confirm the result; however, the nearly identical sequence in the NS1 amplicon area prevented BuV genotyping.

### Generation of recombinant BuV1-3- and TuV-VP2 baculoviruses

Recombinant baculoviruses were created with the Bac-to-Bac Baculovirus Expression System (Invitrogen) according to the manufacturer’s instructions. Briefly, DNA was extracted from the original BuV and TuV DNA-positive stool samples (BuV1 and 2^1^, BuV3^5^ and TuV^2^) with the QIAamp DNA mini kit (Qiagen, Germany). The putative *VP2* gene was amplified with Phusion High-Fidelity DNA Polymerase (Thermo Fischer Scientific) by using primers with designed *BamHI* and SalI restriction sites (Table 4). The amplicon was introduced into the pFastBacDual vector (Invitrogen) by the restriction sites, and the generation of recombinant BuV1-3- and TuV-VP2 bacmids and the subsequent transfection into *Spodoptera frugiperda* Sf9 insect cells were performed according to the manufacturer’s instructions. The resulting virus from each transfection was amplified in three consecutive passages in *Trichoplusia ni* High5 insect cells.

### BuV1-3- and TuV-VP2-VLP expression and purification

For protein expression and VLP production, High5 insect cells were infected with the recombinant baculovirus and grown for 5 days at +27 °C. The cells were collected by centrifugation (4300 g, 5 min) and re-suspended in 20 mM Tris-HCl, pH 8.0, containing Complete-EDTA-free protease inhibitor cocktail (Roche), and were frozen-stored at −20 °C.

The cell suspension was treated with 0.5% sodium deoxycholate (Sigma-Aldrich) for 30 min at +37 °C with 600 rpm shaking, chilled on ice, and sonicated 8 × 10 sec with 50% amplitude. The lysate was centrifuged at 16100 g for 30 min at +4 °C. The supernatant was collected and the remaining pellet was re-suspended, re-sonicated and re-centrifuged. The precleared lysates from the two sonication rounds were combined and loaded onto a CsCl double cushion with densities of 1.52 g/cm^3^ and 1.22 g/cm^3^ in 10 mM Tris-HCl (pH 7.8) containing 1 mM EDTA. Ultracentrifugation was performed at 65 000 *g* for 4 h at 10 °C. Fractions were collected and subjected to SDS-PAGE and the VLP fraction(s) were dialyzed 3 times against PBS. The final purified and dialyzed VLPs were viewed under electron microscopy (Fig. 1).

### BuV and TuV VP2-VLP IgG EIA assays

Various plastics and buffers as well as VLP, antibody and substrate concentrations were systematically compared, and the following protocol was chosen. The purified VP2 VLPs were biotinylated using the EZ-Link Sulfo-NHS-LC-Biotin kit (Thermo Fischer Scientific) according to the manufacturer’s instructions, and the biotinylated VLPs were used as antigens in EIA. Sonicated High5 insect cells were biotinylated as well, and used as control antigen. The biotinylated VLPs or insect cell lysate (80 ng per well in PBS containing 0.05% Tween 20 [PBST]) were immobilized on streptavidin-coated plates (UniverSA96-Lockwell, Kaivogen) for 1 hour at room temperature with 400 rpm shaking. To minimize non-specific background, the plates were post-coated 3 × 10 min with the Diluent (Labsystems Diagnostics). Plasma or serum samples were diluted 1:200 in RED buffer (Kaivogen) and incubated for 1 hour at room temperature with 400 rpm shaking. Wells were washed 4 times with PBST and horseradish-peroxidase conjugated anti-human IgG (DAKO), diluted 1:4000 in the Diluent, was applied for 1 hour. After 4x PBST washes, 3,3′,5,5′-tetramethylbenzidine (TMB, DAKO) was applied for 10 min, the reaction was stopped with 0.5 M H_2_SO_4_, and the ODs were measured at 450 nm (Multiskan EX, Thermo Fischer Scientific). Blank ODs were subtracted from the test ODs.

### IgG testing of human sera

IgG EIA analyses for BuV1, BuV2, BuV3 and TuV as well as for High5 insect cells were carried out in panels, in which each sample was analysed for all five antigens simultaneously in adjacent wells (one well per antigen). This procedure would reveal any unspecific background due to various factors, allowing their assessment in more detail. To confirm the IgG results and determine the antigenic cross-reactivity of BuV1-3 and TuV, all samples showing measurable OD values (≥0.1) were subjected to homologous and heterologous competition, as described for human bocaviruses^19^. In brief, the plasma or serum sample was mixed with an unbiotinylated antigen (VP2-VLP at 20 μg/ml or High5 insect cell lysate at 0.5 mg/ml) prior to the EIA of interest. Each reactive sample was separately blocked both with the homologous antigen, which should fully block a specific reaction, and with all four heterologous antigens, which should not block the specific reaction. The overall BuV-IgG result after competition was considered positive when no heterologous blocking occurred and the residual OD fell upon homologous blocking (i) below OD 0.2, when the non-blocked OD was >0.5; (ii) below OD 0.15, when the non-blocked OD was 0.3–0.5; and (iii) below 50% of the non-blocked OD, when the non-blocked ODs were low, 0.1–0.3. Examples of blocking results are shown in Table 3. For effective blocking in TuV EIA, a 5-fold higher concentration of homologous blocking antigen, compared to that in BuV EIA, was required (Case 18, Table 3).

## Additional Information

**Accession codes**: The VP2 gene sequences of the BuV1-3 and TuV constructs can be found in GenBank, accession nos. KX856937-KX856940.

**How to cite this article**: Väisänen, E. *et al*. Epidemiology of two human protoparvoviruses, bufavirus and tusavirus. *Sci. Rep.* **6**, 39267; doi: 10.1038/srep39267 (2016).

**Publisher’s note:** Springer Nature remains neutral with regard to jurisdictional claims in published maps and institutional affiliations.

## Figures and Tables

**Table 1:** Bufavirus DNA-positive children.

No.	Sample type	BuV DNA quantity (copies per ml supernatant)	Age, gender	Sampling date	Symptoms	Other pathogens found
**1**	Swab	4.6 × 10^3^	20 months, M	27.3.2011	AGE + ARTI	swab: neg* (stool: norovirus, HBoV2# serum: HBoV2#)
**2**	Stool	2.1 × 10^2^	4 yrs 5 months, F	23.2.2010	AGE	norovirus
**3**	Stool	8.5 × 10^2^	23 months, F	6.3.2011	AGE	rotavirus, HBoV2
**4**	Stool	1.6 × 10^3^	10 months, F	9.1.2011	AGE + ARTI	norovirus

^*^Only boca- and coronaviruses were tested from nasal swabs.

^#^An acute bocavirus 2 (HBoV2) infection was diagnosed based on serology in this child^18^. The corresponding stool and serum samples of this child were BuV-DNA negative.

**Table 2:** Bufavirus IgG-positive adults with follow-up samples.

	Geographical background	Sampling date	Absorbance value (OD) without competition				
			BuV1 IgG	BuV2 IgG	BuV3 IgG	TuV IgG	H5 IgG
Case 1	Middle-East	4.3.2010	**2.742**	**2.793**	**2.827**	0.030	0.040
		24.9.2010	**2.657**	**2.958**	**2.958**	0.028	0.042
		22.12.2010	**2.880**	**3.209**	**3.482**	0.026	0.035
		19.7.2011	**2.437**	**2.666**	**2.576**	0.027	0.032
		18.2.2016	**2.683**	**2.419**	**2.675**	0.033	0.046
Case 2	India	6.1.2011	**2.977**	0.032	0.054	0.026	0.036
		19.7.2011	**2.900**	0.039	0.056	0.028	0.038
Case 3	India	10.3.2010	**3.642**	0.040	0.061	0.041	0.037
		6.1.2011	**4.071**	0.025	0.046	0.029	0.024
Case 4	India	11.3.2010	1.924*	0.454*	0.164*	0.079	0.496*
		9.4.2010	2.330*	0.534*	0.228*	0.096	0.616*
Case 5	Finland	19.10.2009	**0.248**	0.069	0.022	0.020	0.050
		1.11.2011	**0.258**	0.084	0.028	0.020	0.055
		21.1.2014	**0.271**	0.091	0.027	0.024	0.057
		17.2.2016	**0.295**	0.126#	0.034	0.029	0.076

The positives are indicated with bolded numbers. H5 = High5 insect cells.

^*^BuV2, BuV3 and H5 reactivity was completely blocked in competition assay by insect cell suspension, but not with the specific BuV VLP. The BuV1 result was blocked only with BuV1. See also Table 3.

^#^BuV2 reactivity was blocked with insect cell suspension, not by BuV2 VLP.

**Table 3:** Examples of competition assay results. Specific blocking is demonstrated with bolded numbers.

Blocking VLP/suspension	Case 1, sample taken 4.3.2010			Case 4*, sample taken 11.3.2010			Case 13	Case 16	Case 18
	Antigen in the well			Antigen in the well			Antigen in the well	Antigen in the well	Antigen in the well
	BuV1	BuV2	BuV3	BuV1	BuV2	BuV3	BuV2	BuV3	TuV
none	2.588	2.761	2.896	2.011	0.644	0.174	0.156	0.142	0.314
BuV1	**0.030**	2.587	2.661	**0.127**	0.458	0.132	0.177	0.107	0.299
BuV2	2.579	**0.038**	2.722	1.949	0.167	0.143	**0.023**	0.111	0.191
BuV3	2.400	2.614	**0.080**	1.904	0.472	0.151	0.191	**0.024**	0.377
TuV	2.513	2.720	2.819	1.997	0.525	0.172	0.164	0.116	**0.101**§
High5 insect cells	2.638	2.845	2.954	2.016	**0.074**^**#**^	**0.087**^**#**^	0.211	0.116	0.401

^*^Case 4 is strongly IgG positive for both BuV1 and insect cells (also seen in Table 2). BuV2 and BuV3 VLPs have been shown to have insect cells impurities (data not shown), which causes more reactivity towards BuV2 and BuV3 antigens. This reactivity is completely blocked by insect cell suspension (marked with^#^), and the sample is therefore considered to be IgG positive only for BuV1 among the tested viral antigens. The blocking effect of BuV2 VLP towards BuV2 is interpreted to be caused by the insect cell impurities rather that the VLP itself.

^§^5x more blocking antigen was used to block the IgG reactivity.

**Table 4:** Primers used for amplifying the *VP2* gene of BuV1, 2 and 3 and TuV.

Primer	Sequence
BuV1 VP2 fwd BamHI	TAggatccATGACTGACACACAAGATGTATCTGA
BuV1 VP2 rev SalI	ATTgtcgacTCCATTTTAGATTGTGTAGTTAGGCATAC
BuV2 VP2 fwd BamHI	TAggatccATGTCTGAAAGCAATGAAATTGGAG
BuV2 VP2 rev SalI	ATTgtcgacTTACATTGTGTAGTTAGGCATGGCTCT
BuV3 VP2 fwd BamHI	TAggatccATGTCCGAAAGCAATGAAATTGACG
BuV3 VP2 rev SalI	ATTgtcgacTTAGTATGTGTAGTTTGGCATTGCTC
TuV VP2 fwd BamHI	TAggatccATGGCAGCCTCTAGCTCAGACAGTG
TuV VP2 rev SalI	ATTgtcgacTTAGTAAACAGTAGAGGGTACAATTCTTC
